# A preliminary study of the efficacy of bimodal versus unimodal stumble recovery responses in a powered knee prosthesis

**DOI:** 10.1017/wtc.2026.10040

**Published:** 2026-04-07

**Authors:** Shane T. King, Maura E. Eveld, Leo G. Vailati, Karl E. Zelik, Michael Goldfarb

**Affiliations:** 1 Mechanical Engineering, Vanderbilt University, USA; 2 Electrical Engineering, Vanderbilt University, USA; 3 Biomedical Engineering, Vanderbilt University, USA; 4 Physical Medicine & Rehabilitation, Vanderbilt University, USA

**Keywords:** prosthetics, rehabilitation robotics, control, biomechatronics, biomechanics

## Abstract

Transfemoral prosthesis users demonstrate a higher fall rate due to tripping than able-bodied controls in previous laboratory studies. In particular, early swing demonstrates the greatest disparity, where able-bodied controls typically utilize an elevating strategy to cross the obstacle in the same stride that the perturbation occurs, rather than the lowering strategy, where swing is ended prematurely and the obstacle is crossed in the following stride. However, due to the passive nature of most commercial knee prostheses, the elevating strategy is largely inaccessible to prosthesis users, potentially contributing to the increased fall rate in early swing. To investigate the effects of reintroducing the elevating strategy to transfemoral prosthesis users, a bimodal stumble recovery controller was developed for a powered knee prosthesis that utilized the elevating and lowering recovery strategies, selected based on the post-impact kinematics of the prosthesis. The Bimodal controller was compared to a unimodal controller that only used the lowering strategy. Three transfemoral prosthesis users underwent a series of treadmill-based obstacle perturbations with each controller following an acclimation period. All participants successfully used the elevating response in the early swing phase. On average, the elevating response reduced the disturbance to participants’ trunk kinematics and the reliance on harness support. While the Bimodal controller sometimes resulted in a recovery strategy mismatch for two participants, the mismatch still resulted in outcome metrics comparable to the unimodal controller. Overall, results suggest that the inclusion of the elevating and lowering strategies may improve stumble recovery outcomes for some transfemoral prosthesis users.

## 1. Introduction

Prior survey and experimental studies demonstrate that transfemoral prosthesis users experience a higher fall risk compared to able-bodied individuals (Talbot et al., 2005; Hafner et al., 2007; Kahle et al., 2008; Verma et al., 2016; King et al., 2019; King et al., 2024), with 47% of transfemoral prosthesis users also reporting a fear of falling (Miller et al., 2001). Able-bodied individuals employ a set of robust recovery reflexes that largely prevent falls following a trip or stumble. A previous experimental study demonstrated a 100% recovery rate in an able-bodied cohort following a series of treadmill-based obstacle perturbations while walking at 1.1 m/s (King et al., 2019). Meanwhile, transfemoral prosthesis users are much more constrained in their ability to respond to a stumble, partially due to the passive prosthetic knee joint limiting their responses, and therefore, their ability to successfully recover. Another previous experimental study reported that transfemoral prosthesis users had only a 46% recovery rate to treadmill-based obstacle perturbations while walking at 0.8 m/s (King et al., 2024). It is noteworthy that both studies with the able-bodied and transfemoral prosthesis user cohorts employed the same experimental apparatus.

Able-bodied individuals employ three primary recovery strategies in response to a stumble: elevating, lowering, and delayed lowering. In the elevating strategy, the foot is lifted over the obstacle in the same swing phase as the perturbation by flexing the knee and hip (Eng et al., 1994; King et al., 2019). In the lowering strategy, the foot is lowered to the ground before crossing the obstacle (i.e., swing is terminated prematurely), and the obstacle is cleared in the subsequent swing phase (Eng et al., 1994; King et al., 2019). The elevating strategy is well-suited for early swing stumbles due to the high forward swing limb momentum following toe off, while the lowering strategy is well-suited for late swing stumbles due to the diminished forward swing limb momentum preceding heel strike. The ability to employ two recovery actions is referred to herein as “bimodal.” The third strategy, delayed lowering, is a transitionary strategy between the elevating and lowering strategies where the foot is initially elevated but subsequently lowered (King et al., 2019; Schillings et al., 2000). Due to the transitionary nature of the response and the final behavior of the strategy ultimately being that of the lowering strategy, delayed lowering is considered a variation of lowering in this work.

Although studies have shown that transfemoral prosthesis users attempt to use both the elevating and lowering recovery strategies (Crenshaw et al., 2013b; Shirota et al., 2015; King et al., 2024), they are far less effective at avoiding a fall than able-bodied individuals (King et al., 2019; Eveld et al., 2021), presumably because replicating the able-bodied strategies requires power at the knee joint (i.e., action), while typical knee prostheses are energetically passive (i.e., capable only of reaction). The elevating strategy is particularly affected since prostheses have no ability to provide active knee flexion immediately after a perturbation, which restricts the prosthesis user’s ability to utilize the response (King et al., 2024). In the case where the elevating strategy cannot be used in the early swing, prosthesis users are left with the lowering strategy as their only option, which is not the biological norm in the early swing phase and is challenging to perform successfully due to the need for a complete reversal of swing limb forward momentum in a short time period.

Physically, the elevating strategy is effective in the early swing phase as it preserves the forward momentum of the swing limb following the recent toe-off rather than requiring a reversal of momentum to lower (Eng et al., 1997). Additionally, since the elevating strategy occurs in early swing, the contralateral limb is in early stance, such that the body’s center of mass (CoM) can continue to be supported for a sufficient period to allow the swing limb to clear the obstacle and extend the base of support within the same stride (Eng et al., 1994). As such, the elevating response capitalizes on the body’s dynamic state following a perturbation in the early swing phase to reduce the effort and number of strides required to return to a fully supported state.

Prior studies have also described limitations in the stumble recovery response of passive prostheses beyond restricting the use of the elevating strategy. Knee buckling at ground contact due to a combination of inadequate stance support and swing extension assistance is a common cause of falls (Crenshaw et al., 2013b; King et al., 2024). Additionally, an inability to initiate swing phase with the prosthetic limb following a perturbation due to imbalance frequently results in a fall due to delayed recovery or from the foot remaining stuck behind the obstacle (King et al., 2024).

Recently, powered knee prostheses have emerged (e.g., Sup et al., 2009; Rouse et al., 2013; Lawson et al., 2014; Elery et al., 2018; Lenzi et al., 2018; Lee et al., 2019; Tran et al., 2019; Bartlett et al., 2022; Culver et al., 2022) that are capable of addressing the stumble recovery shortcomings present in passive prosthetic limbs, including replicating the recovery responses employed by able-bodied individuals to a much greater extent than passive knee prostheses. Reintroducing the elevating strategy to prosthesis users through added power in the prosthetic swing phase could potentially reduce the likelihood of falls for transfemoral prosthesis users.

Previous efforts aimed at reducing fall likelihood in the transfemoral prosthesis user population have primarily focused on passive resistive prosthesis behaviors to limit the magnitude of the perturbation (Hafner et al., 2007; Kahle et al., 2008; Blumentritt et al., 2009; Highsmith et al., 2010; Fuenzalida Squella et al., 2018) and compensatory step training to expose and condition individuals to stumble scenarios to improve their ability to respond (Crenshaw et al., 2013a). Recent investigations with powered mechatronic interventions have focused on obstacle crossing and avoidance (Gordon et al., 2019; Rezazadeh et al., 2019; Thatte et al., 2019; Mendez et al., 2020; Cheng et al., 2023; Chen et al., 2024; Zhu et al., 2025) in an effort to reduce the incidence of obstacle perturbations. While obstacle avoidance is a critical aspect required to mitigate prosthesis user fall risk, providing prosthetic behaviors that actively assist in the recovery from a stumble is an important element of fall mitigation that has not been well addressed. This work experimentally examines the effect of implementing bimodal stumble recovery behaviors in a powered knee prosthesis (with regard to potentially reducing falls in response to a stumble perturbation), relative to employing a unimodal, lowering-only behavior instead.

Specifically, this paper describes a controller for a knee prosthesis that includes both elevating and lowering stumble recovery responses; implements the controller on a powered knee prosthesis prototype; and conducts a study involving three transfemoral prosthesis users that compares their ability to recover from stumble perturbations with a bimodal recovery response versus a unimodal, lowering-only recovery response. This study attempts to address four fundamental questions concerning the implementation of a bimodal stumble recovery response. (1) Can prosthesis users utilize the elevating strategy? With the stumble recovery response being a reflexive one, is it possible to reintroduce a strategy that the user has not had direct access to for some time? (2) Does the elevating strategy provide a benefit relative to the lowering strategy in the early swing for prosthesis users? (3) Can the prosthetic device coordinate with the user’s response to a stumble in a consistent manner? (4) Does having two potential recovery responses lead to increased uncertainty and make recovery more challenging for users?

## 2. Methods

### 2.1. Experimental knee prosthesis prototype

**Figure 1.**
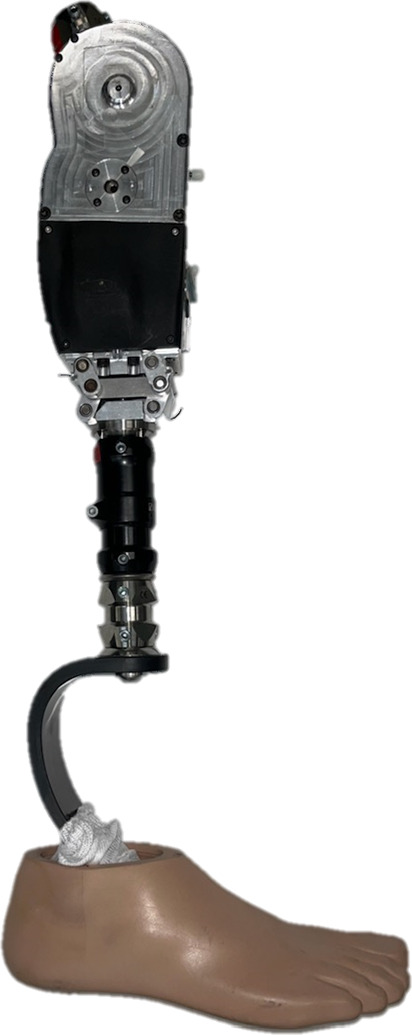
Image of the Vanderbilt powered knee prosthesis used in this work.

The controller and study described herein were implemented using the Vanderbilt powered knee prosthesis prototype, shown in Figure 1. The prosthesis uses a brushless direct current motor (EC-4 pole 30 mm 36 V, Maxon, Sachseln, CHE) with a three-stage belt-chain transmission and a gear ratio of 176:1. Its maximum torque at the knee is ~85 Nm, and the total knee range of motion is 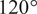 . The knee prosthesis is powered with a nominally 40 V lithium-ion battery pack. The onboard sensing includes an incremental motor encoder, an absolute knee encoder, a 6-axis inertial measurement unit (IMU), and a full-bodyweight range axial load cell. Sensor fusion of the motor and knee encoders provides the knee angle for the control system, while sensor fusion of the IMU’s accelerometer and gyroscope signals provides the shank angle for the control system. The thigh angle is computed by the combination of knee and shank angle measurements. Low-level motor control is performed onboard the prosthesis on its embedded system, while the high-level walking and stumble recovery controllers are performed on a separate laptop using Simulink Desktop Real-Time (Mathworks, Natick, USA) that uses a controller area network interface to communicate with the prosthesis. For more information about the powered prosthesis, see Lawson et al. (2014).

### 2.2. Walking controller

**Figure 2.**
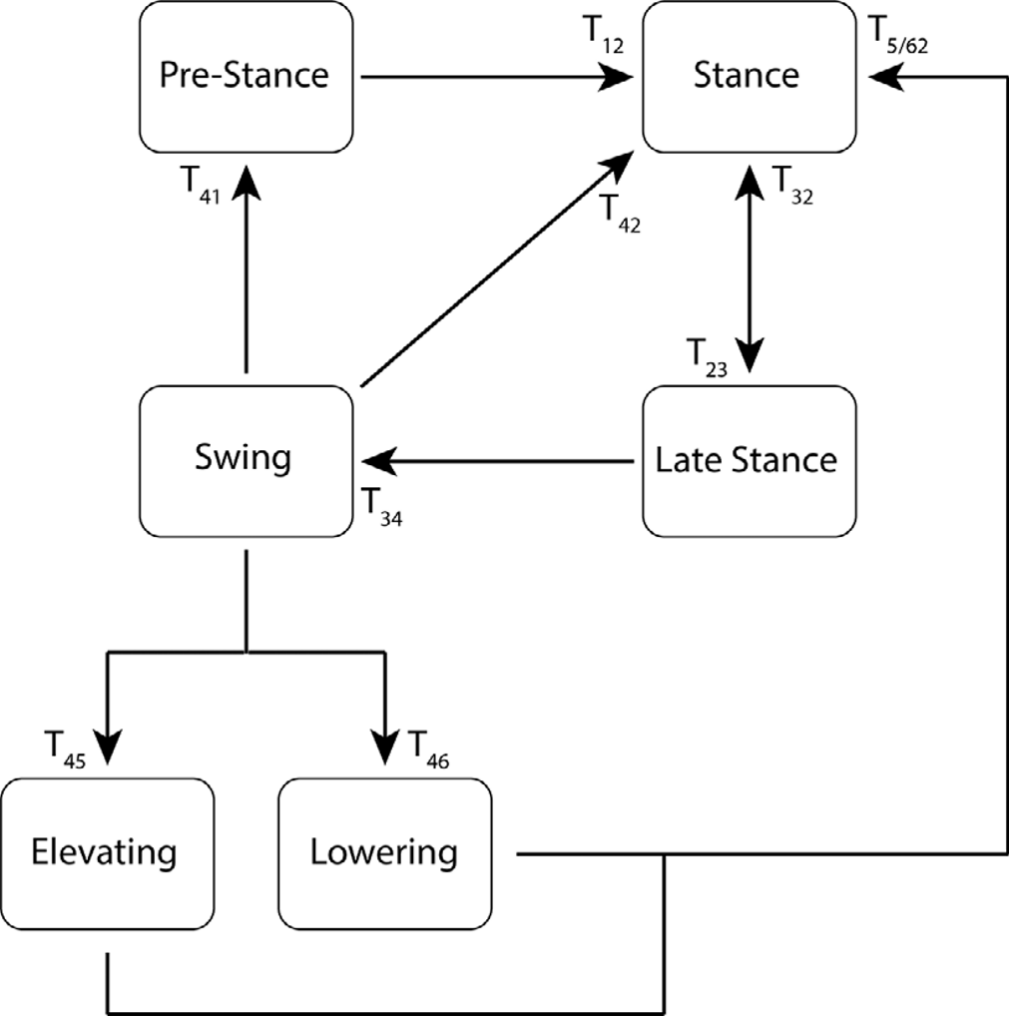
Walking and stumble recovery finite state machine. Transitions are provided in Tables 1 and 2.

The stumble recovery responses were implemented around a nominal powered prosthesis walking controller. The walking controller finite state machine (FSM), shown in Figure 2, consists of four states: Pre-Stance, Stance, Late Stance, and Swing. Transitions are defined in Table 1. Transition thresholds are representative, as some tuning is required to adjust for participant weight, height, prosthetic alignment, and comfort/preference. Pre-Stance, Stance, and Late Stance all use the impedance control equation:
(2.1)

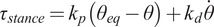

where 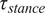 is the commanded torque at the knee during the stance states, 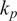 is the proportional gain, 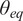 is the desired stance equilibrium knee angle, 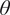 is the measured knee angle, 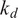 is the derivative gain, and 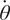 is the measured knee angular velocity. The Pre-Stance state is in effect when the swing trajectory has completed, but the leg has not yet made ground contact to ensure the prosthesis is prepared to provide stance support at heel strike. Relative to Stance, Pre-Stance uses reduced proportional and derivative gains to prevent oscillation before heel strike. Stance uses increased gains to ensure robust stance support (where oscillation is not an issue due to high environmental impedance). Late Stance allows for knee flexion by setting the proportional gains to zero to prepare for entering the swing phase. However, a high derivative gain in the knee extension direction is maintained for stability in the event of a misstep or loss of balance. Friction compensation is utilized to allow for easier flexion without needing to overcome the intrinsic friction of the device. Knee yielding in the Late Stance state not only allows for a more comfortable transition to swing for users, but it also better allows the thigh to establish the kinematic initial conditions associated with the swing phase. Finally, Swing uses proportional-derivative trajectory control using the equation below:
(2.2)

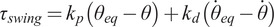

where 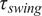 is the commanded torque at the knee during the swing state, and all other parameters are as presented in Eq. 2.1, with the addition of 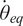 , which is the desired knee angular velocity along the trajectory. The trajectory is nominally a cadence-adaptive spline that is generated based on able-bodied knee kinematics with peak flexion of 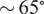 (varied slightly across participants for their preference, comfort, and symmetry) and matched to the initial angle and angular velocity conditions at the transition into swing. While the temporal duration of the spline can be adjusted by cadence between strides, during a given swing phase, progression through the trajectory is directly temporally controlled. Proportional and derivative gains are defined separately for both swing flexion and swing extension.

**Table 1.** Walking FSM state transition conditions. Force transitions displayed using a representative body weight of 80 kg.

Transitions	Conditions
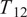 : Pre-Stance 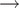 Stance	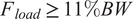
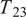 : Stance 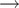 Late Stance	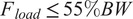 **AND**
	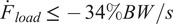 **AND**
	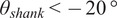 (ignored after perturbation)
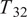 : Late Stance 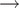 Stance	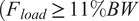 **AND** 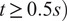 **OR**
	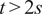 **OR**
	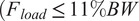 **AND** 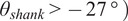 (ignored after perturbation)
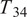 : Late Stance 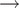 Swing	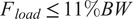 **AND**
	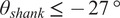 (not applied in the step after perturbation)
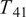 : Swing 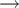 Pre-Stance	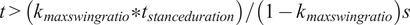 **OR**
	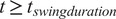
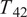 : Swing 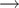 Stance	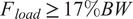

**Table 2.** Stumble recovery FSM state transition conditions. Force transitions displayed using a representative body weight of 80 kg

Transitions	Conditions
Perturbation detection	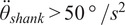 **OR**
	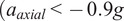 **AND** 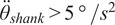 **AND** 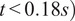
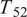 : Elevating 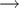 Stance	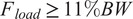
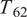 : Lowering 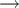 Stance	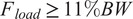
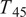 : Swing 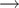 Elevating	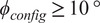 **OR**
	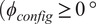 **AND** 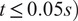
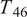 : Swing 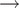 Lowering	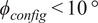 **OR**
	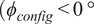 **AND** 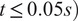

For a typical gait cycle, the transition from Pre-Stance to Stance is determined by loading the prosthesis, the Stance to Late Stance transition is determined by initial unloading of the prosthesis and the forward lean of the shank, the Late Stance to Swing transition is determined by full unloading and further forward rotation of the prosthesis, and the Swing to Pre-Stance transition is determined by completion of the swing trajectory or a timeout in the event of an obstruction. Alternatively, the Swing to Stance transition is determined by loading the prosthesis if done so before the completion of the swing trajectory. Additionally, a safety transition from Late Stance back into Stance is in place in the event that any of the following occur: the unloading threshold is not reached (i.e., if the user begins loading the leg once again), the forward tilt threshold is not reached (i.e., if the user stops tilting forward before lifting the leg up, such as in nonwalking stepping), or a timeout to prevent excessive or uncontrolled knee yielding during a misstep.

### 2.3. Stumble recovery controllers

#### 2.3.1. Perturbation detection

Stumble perturbations are detected with the combination of the gyroscope and the accelerometer from the 6-axis IMU. Due to the varying nature of the perturbation impulse and orientation of the foot across the swing phase, different conditions were used to detect perturbations while the knee was in extension versus flexion. In flexion during the first portion of the swing phase, the perturbation impulse is lower in magnitude due to the knee lifting the toe up while the obstacle makes contact on the top of the toe. As such, a perturbation is detected when a threshold of axial linear acceleration (as measured by the accelerometer) is exceeded. Additionally, two guard conditions are present; a low guard threshold on shank angular acceleration must be exceeded to avoid false positives from inadvertent ground contact (e.g., a scuff) and a time guard condition must not be exceeded to constrain this type of perturbation detection to early and mid-swing phase, avoiding false positives during the transition from swing to stance at heel strike. In swing extension during the latter portion of the swing phase, the perturbation causes a large impulse to the front of the toe because the knee is accelerating the foot into the obstacle. As such, a perturbation is detected when a high threshold of shank angular acceleration (as measured by the time derivative of the gyroscope signal in the sagittal plane) is exceeded. While the nature of the perturbation impact varies with knee velocity, knee velocity is not used in the detection conditions to allow for overlap in detection conditions during the transition from swing flexion to swing extension for improved reliability. The detection conditions were selected experimentally by observing the response of all kinematic measurements and their time derivatives during a large number of walking trials, with and without stumble perturbations, during pilot data collection. Thresholds were selected based on parameters that reliably detected perturbations while avoiding false positives during nonperturbed strides. Thresholds are detailed in Table 2.

#### 2.3.2. Recovery strategy decision

**Figure 3.**
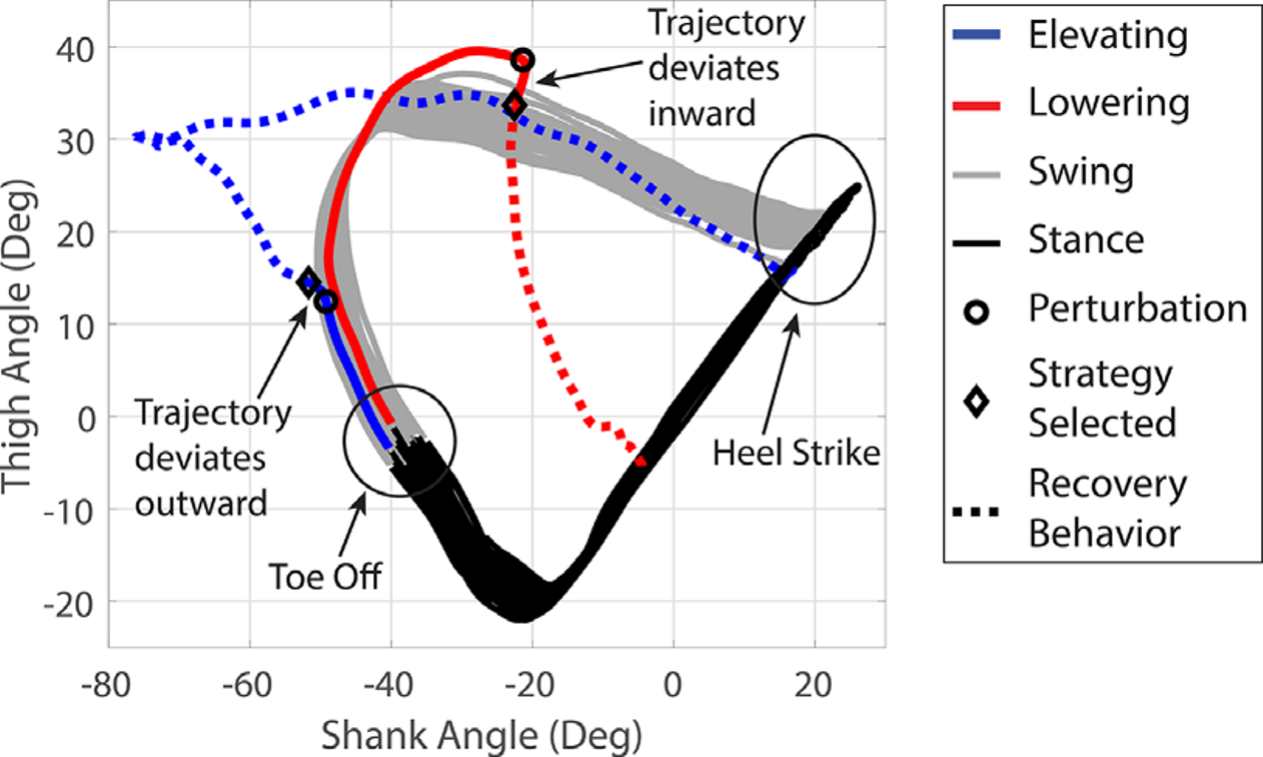
Thigh-shank angular configuration space recovery strategy decision algorithm. Shank angle is plotted on the *x*-axis in degrees, and thigh angle (summation of shank angle and knee angle) is plotted on the *y*-axis in degrees. The loop is the standard configuration space during a normal stride, with stance in black and swing in gray, with several steady state strides depicted. The loop path moves clockwise with time. The blue line is an example of an elevating strategy, and the red line is an example of a lowering strategy. The solid portion of the line is the swing phase before the recovery strategy transition, and the dashed line is the portion following the transition. Perturbation detection is shown with a black circle, and the recovery strategy decision is shown with a black diamond.

In the case of the Bimodal stumble recovery controller, the controller must decide which of the two responses, elevating or lowering, should be implemented following perturbation detection. The decision strategy employed here was inspired by the observation of experimental biomechanical data from a previous able-bodied cohort study (King et al., 2019; Eveld et al., 2021) and utilized the collision dynamics immediately following the perturbation to reinforce the natural movement resulting from the collision between the foot and the obstacle. The relevant, measurable collision dynamics can be represented in a configuration space of sagittal plane shank angle versus sagittal plane thigh angle, which are measured relative to the vertical by the IMU or by a combination of the IMU and knee angle measurement (via encoder data), respectively. It is noteworthy that sagittal is defined herein as the plane orthogonal to the prosthetic knee joint axis, and therefore is not strictly sagittal in a global frame. Figure 3 shows measured data from the knee prosthesis during several unperturbed walking strides, where the black traces represent the stance phase, and the gray traces represent the swing phase. The red trace shows a representative trajectory corresponding to a lowering strategy, while the blue shows a representative trajectory corresponding to an elevating strategy. The perturbation detection is indicated in both cases by a black circle, while the moment at which a recovery strategy decision is made is indicated by a black diamond. The segments between the two shapes are the natural collision dynamics before a decision regarding the bimodal response. In the case of a lowering response, the natural collision dynamics direct the trajectory toward the interior of the swing-phase elliptical path, while the natural collision dynamics corresponding to an elevating response direct the trajectory toward the exterior. As such, the controller observes the direction of the trajectory deviation following the collision, either interior or exterior to the ellipse, and then chooses a knee trajectory that reinforces the initial movement: a lowering response if the collision moves the trajectory interior, and an elevating response if the collision moves the trajectory exterior. The respective response trajectories for each strategy are shown as dashed lines in Figure 3.

The controller implementation employs a 20 ms delay between perturbation detection and recovery strategy decision (i.e., it observes the collision dynamics for 20 ms before making a decision). During the 20 ms decision window, the swing controller continues to follow the unperturbed knee angle trajectory in an effort to avoid imparting potentially disruptive changes in momentum as a result of stopping and restarting movement of the knee during swing. It should also be noted that the controller assumes the default response to be a lowering response, and therefore checks only for a collision dynamic that corresponds to an elevating response (i.e., movement external to the ellipse). Furthermore, the movement external to the ellipse must meet or exceed a threshold to elicit an elevating response. In this implementation, a 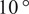 external angular offset threshold with respect to the original unperturbed trajectory was used (see Table 2). During potentially ambiguous perturbations with small configuration space deviation angles, the offset was implemented to bias the decision toward the more familiar lowering strategy in an effort to improve the likelihood of a successful recovery. The 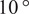 value was selected based on pilot testing results. The angular offset is measured as the Euclidean norm from the unperturbed, elliptical configuration space trajectory, which is measured as a 4 ms window average before the perturbation. As such, the controller always selects a lowering response, unless the collision dynamics deviate from the trajectory at least 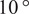 outside the elliptical path 20 ms after the collision, in which case it selects an elevating response. In very early swing (i.e., <0.05 s from toe off), the angular threshold is 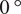 because a lowering strategy is less likely to be adopted very early in swing.

It is important to note that the 20 ms observation window was selected to ensure accurate measurement of the post-impact dynamics in order to provide consistent recovery strategy selection for similar magnitude perturbations while remaining as short as possible. At the current timescale, the observation window is shorter than the shortest observed polysynaptic reflexive responses during stumble recovery (60 ms), as faster monosynaptic responses have not been observed (Eng et al., 1994). Additionally, the window is significantly shorter than the potential volitional response onset (>160 ms) (Eng et al., 1994). The short observation window ensures only the post-impact dynamics play a role in the recovery strategy decision in an effort to facilitate user coordination with the elevating strategy by reinforcing the movement before the onset of the reflexive response, especially since the elevating strategy is not typically a response that transfemoral prosthesis users can feasibly use with their prescribed prostheses. The specific 20 ms value was selected based on consistent strategy selection performance during pilot testing.

#### 2.3.3. Stumble recovery state machine

**Figure 4.**
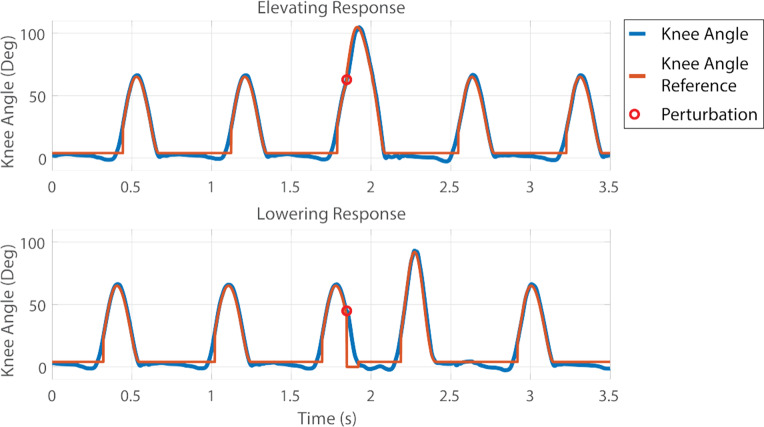
Examples of commanded and actual knee angle trajectories for the walking and stumble recovery controller states. The elevating strategy is shown on the top, and the lowering strategy is shown on the bottom. The knee angle from the prosthesis is shown in blue, while the reference knee angle is shown in orange. The moment of the perturbation is depicted with a red circle.

To implement the stumble recovery responses, two additional states were added to the walking controller, both illustrated in Figure 2. The elevating state suspends the swing state trajectory and appends it with a new spline modeled after able-bodied elevating responses, causing the knee to flex further than in typical swing, 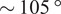 , before quickly extending to prepare for the stance phase in 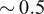 s (both parameters were adjusted slightly based on user preference and performance during an acclimation phase). The lowering state also suspends the swing state trajectory and extends the leg to 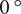 , essentially assuming the Stance state. Additionally, the lowering state triggers an increased peak knee flexion spline in the subsequent swing state 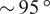 (also adjusted slightly based on user preference and performance during acclimation) to assist in obstacle clearance in the following step. In the elevating state, if the obstacle is not successfully crossed (based on a negative shank angle measurement), the same trigger occurs to increase the peak knee flexion angle in the subsequent swing state. Both of the stumble recovery states can only be entered from the swing state after a perturbation has been detected and a decision has been made (in the case of the Bimodal controller). Examples of representative recovery strategy swing trajectories, shown relative to the trajectories employed in unperturbed strides, are displayed in Figure 4. The elevating state features greater knee flexion than the lowering state due to improved initial conditions, allowing for increased flexion in the appropriate time span for the recovery. The increased flexion provides greater toe clearance during a highly variable response, emulating the behavior observed in able-bodied stumble recovery responses, which typically employ excessive knee flexion to ensure obstacle clearance during high magnitude perturbations during the early and mid-swing phase (King et al., 2019). The lowering state features increased knee extension to provide an increased stance knee support moment arm at initial ground contact when knee buckling risk is highest.

### 2.4. Experimental design

Experiments were conducted to examine the efficacy of the proposed Bimodal controller and to determine the extent to which users might accept or coordinate with the Bimodal controller. Experiments were also conducted employing a unimodal controller that always resorted to a lowering strategy, herein referred to as the Lowering Only controller. The latter was intended as a highly predictable behavior to contrast the potential uncertainty associated with the bimodal response. Three participants were enrolled in the study. Table 3 lists the respective demographic information of each. The experimental design followed a similar format to previously published stumble perturbation studies (King et al., 2019, 2024; Eveld et al., 2021; Eveld et al., 2022), although with the addition of acclimation periods between testing periods to allow participants time to adapt to the new controllers. First, participants underwent a fitting for the powered knee prosthesis and an acclimation period for walking and the Lowering Only controllers; followed by testing with the Lowering Only controller; followed by an acclimation period for the Bimodal controller; and finally followed by testing with the Bimodal controller. The testing and acclimation days are described in the respective sections below. The Lowering Only controller was tested first as it was most similar to what the participants were familiar with on their passive, daily-use prostheses, thus minimizing the required initial acclimation time. The experimental protocol was approved by the Vanderbilt University Institutional Review Board. All participants gave their written informed consent.

**Table 3.** Participant demographic information

Participant	Age	Sex	Prosthetic side	Etiology	Years of prosthesis use	Prescribed prosthesis
Participant 1	32	Male	Left	Trauma	4	Blatchford KX06
Participant 2	28	Female	Right	Congenital	27	Ottobock C-Leg 4
Participant 3	62	Male	Right	Trauma	49	Ottobock C-Leg 4

#### 2.4.1. Experimental protocol

The experimental protocol consisted of each participant walking on a force-instrumented, split-belt treadmill (Bertec, Columbus, USA) at 0.8 m/s. After a randomized number of strides between 20 and 45, an obstacle was deployed at a specific point in the swing phase via a custom obstacle perturbation system using a predictive targeting algorithm to produce precisely timed perturbations (King et al., 2019). The obstacle used in this study was a 16 kg (35 lb) steel block that measured 20 cm wide, 12.5 cm long, and 7.5 cm high (8.125” × 5” × 3”). Targeted swing percentages included 25–50% in 5% increments, as well as 75% for the prosthetic limb. Additional perturbations at 25, 50, and 75% for the sound limb were included to act as catch trials to reduce potential anticipation by participants. Additional trials were performed in the case of a mistrial or due to poor perturbation targeting. In addition to the 10 trials, a warm-up period of randomized early and late swing prosthesis-side perturbations was performed for each participant. The warm-up period ensured each participant was comfortable and re-acclimated to the powered prosthesis that day. At least three trials were performed during the warm-up period, but up to 10 trials were performed if some adjustment to the prosthesis or controller was needed based on participant comfort feedback. Any warm-up trials that were performed after the final adjustments were included in the final data set to increase repeated measures. The concentration of early prosthesis-side perturbations was chosen to allow for increased data points in the region of swing where the two stumble recovery controllers functioned differently, as well as to capture the transition period between the elevating and lowering strategies for the Bimodal controller. Perturbation timing was randomized for all trials during the experiment.

During the perturbation trials, participants wore sensory occlusion gear, including earbuds with white noise, passive noise-cancelling headphones, and dribble goggles that block the inferior visual field. Serial Sevens (i.e., counting backwards by sevens from an initial seed number) was performed as a distraction task to prevent compensatory behavior. Visual feedback was provided via video monitor to keep participants centered on the treadmill. Handrails were removed from the treadmill, but participants wore a load cell-instrumented, full-body harness, which monitored how much bodyweight support was provided to the participant during a trial to determine if a fall had occurred. Full-body, infrared motion capture (Vicon, Oxford, GBR) and 6-degree-of-freedom force plate data were collected during all trials at 200 and 1,000 Hz, respectively. Full-body motion capture included foot, shank, thigh, waist, torso, upper arm, and forearm segments. A video of representative trials for each participant, controller, and recovery strategy is included with the Supplementary Material of this work.

#### 2.4.2. Acclimation protocol

The experiment required an acclimation period to allow the participants to adapt to the two stumble recovery controllers (Bimodal and Lowering Only). The Bimodal controller and the elevating response in particular were quite different from the typical response of a passive prosthesis. Before either testing day, at least 1 session (each ~2 hr in duration) was spent to allow the participant to acclimate to the stumble recovery controller at hand. The acclimation protocol included allowing the participant to intentionally stumble on an obstacle while holding the handrails so they could watch and feel how the prosthesis would respond in both early and late swing perturbation scenarios (whether that be elevating versus lowering or simply lowering every time). Elements of the experiment protocol were then added one at a time as the participant became comfortable with the previous step. First, the sensory occlusion equipment was introduced, then the perturbation timing was randomized, then the participant was instructed to use only a single handrail, followed by no handrails, and then the handrails were removed entirely. Once a participant was able to consistently respond to perturbations of randomized timing without handrails, the trials moved from the acclimation phase to the data collection phase. A consistent response was defined as the user attempting to perform the same or similar recovery strategies to multiple (at least three) similarly timed perturbations. It is noteworthy that these recovery attempts did not always have to be successful to be deemed consistent. Participants required between one and three acclimation sessions to adjust to each controller.

### 2.5. Data processing and analysis

Gait kinematic and kinetic data were computed from the motion capture and force plate data via Visual 3D inverse dynamics software (C-Motion, Germantown, USA). Force plate and motion capture data were filtered with a zero-phase, third-order, low-pass Butterworth filter with a cutoff frequency of 15 and 6 Hz, respectively. Data analysis was performed in MATLAB (Mathworks, Natick, USA). Perturbation swing percentage was determined via a spike in the anterior–posterior force plate data. Since actual falls were precluded by a load cell-instrumented harness, falls were instead defined using a discrete threshold on harness assistance. Any trial with an impulse (as measured by the load cell-instrumented harness) of 50% bodyweight seconds or greater was defined as a fall. Harness assistance of >10% bodyweight seconds was classified as harness assist rather than a fall. Falls and harness assists are collectively referred to as failed recoveries. In the event of force plate crossover steps following the perturbation, gait events were determined using vertical foot CoM velocity (O’Connor et al., 2007).

In addition to harness impulse, trunk kinematic metrics were also computed to better characterize the quality of the recovery for each trial and to indicate an overall likelihood of a real-world fall. The trunk metrics include peak trunk angle and peak trunk angular velocity during the recovery response (limited to within the first two recovery steps on the prosthesis side) relative to the value at the perturbation. These metrics have been used in previous studies to indicate overall quality of recovery (Pavol et al., 2001; Crenshaw et al., 2012; Eveld et al., 2022; King et al., 2024) as they describe the amount of disturbance to the torso of the participant, which has a major effect on the CoM support. The trunk kinematic metrics allow for a continuous measure of the quality of each recovery rather than the discrete measure that fall rate thresholds provide. All statistical analyses on these metrics used the nonparametric Wilcoxon rank-sum test with the baseline threshold for statistical significance defined at *p* < .05. To account for comparisons across multiple metrics, a Bonferroni correction was applied, bringing the threshold for statistical significance to *p* < .017.

## 3. Results

**Figure 5.**
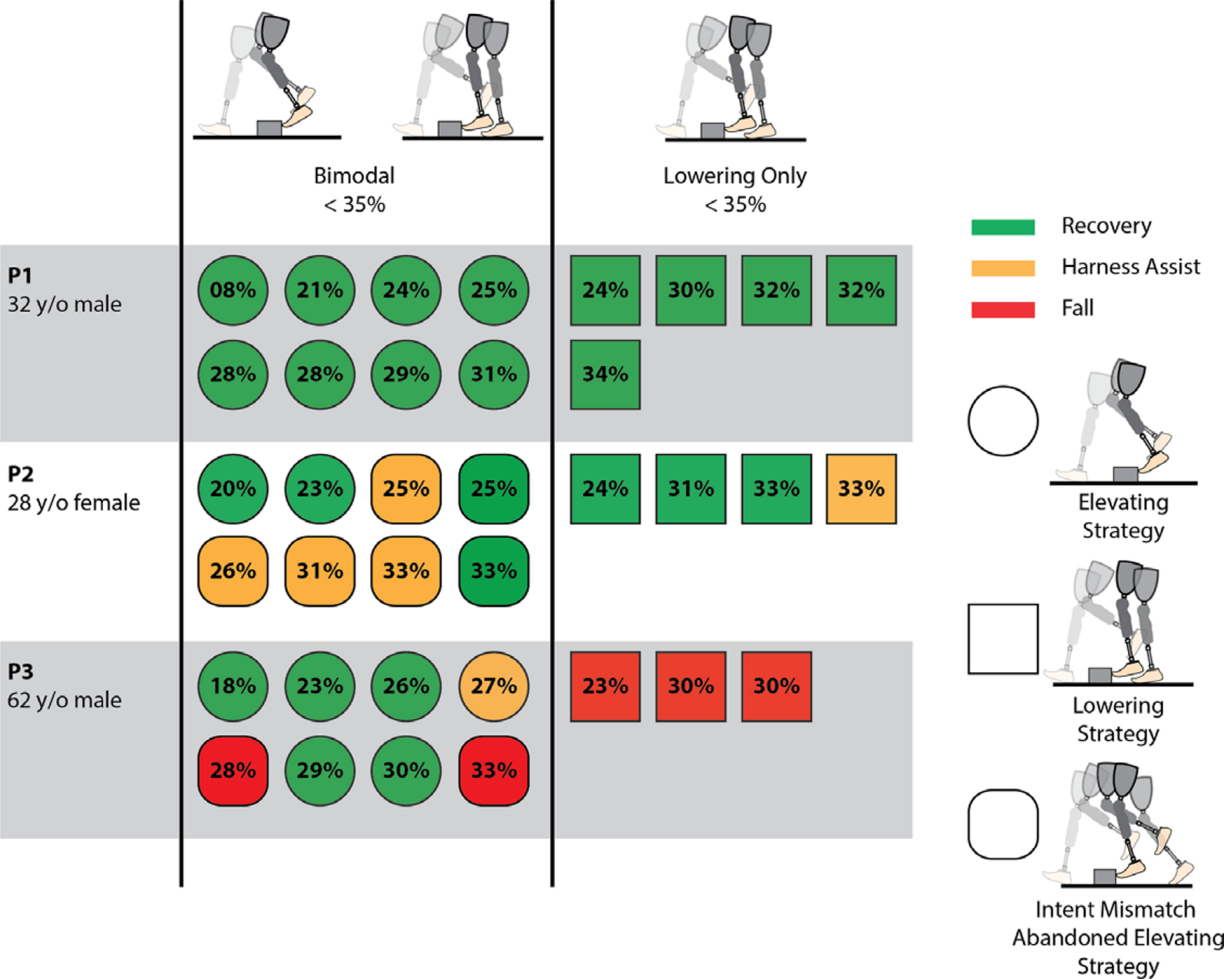
Early swing recovery outcomes (<35% swing phase) for each participant and controller. Green indicates a recovery, yellow indicates a harness assist, and red indicates a fall. Elevating strategies are depicted by a circle, lowering and delayed lowering strategies are depicted by a square, and intent mismatch trials where the elevating response was abandoned after being chosen by the prosthesis are depicted by rounded squares. The percentage of the swing phase of each perturbation is indicated on the shape.

**Figure 6.**
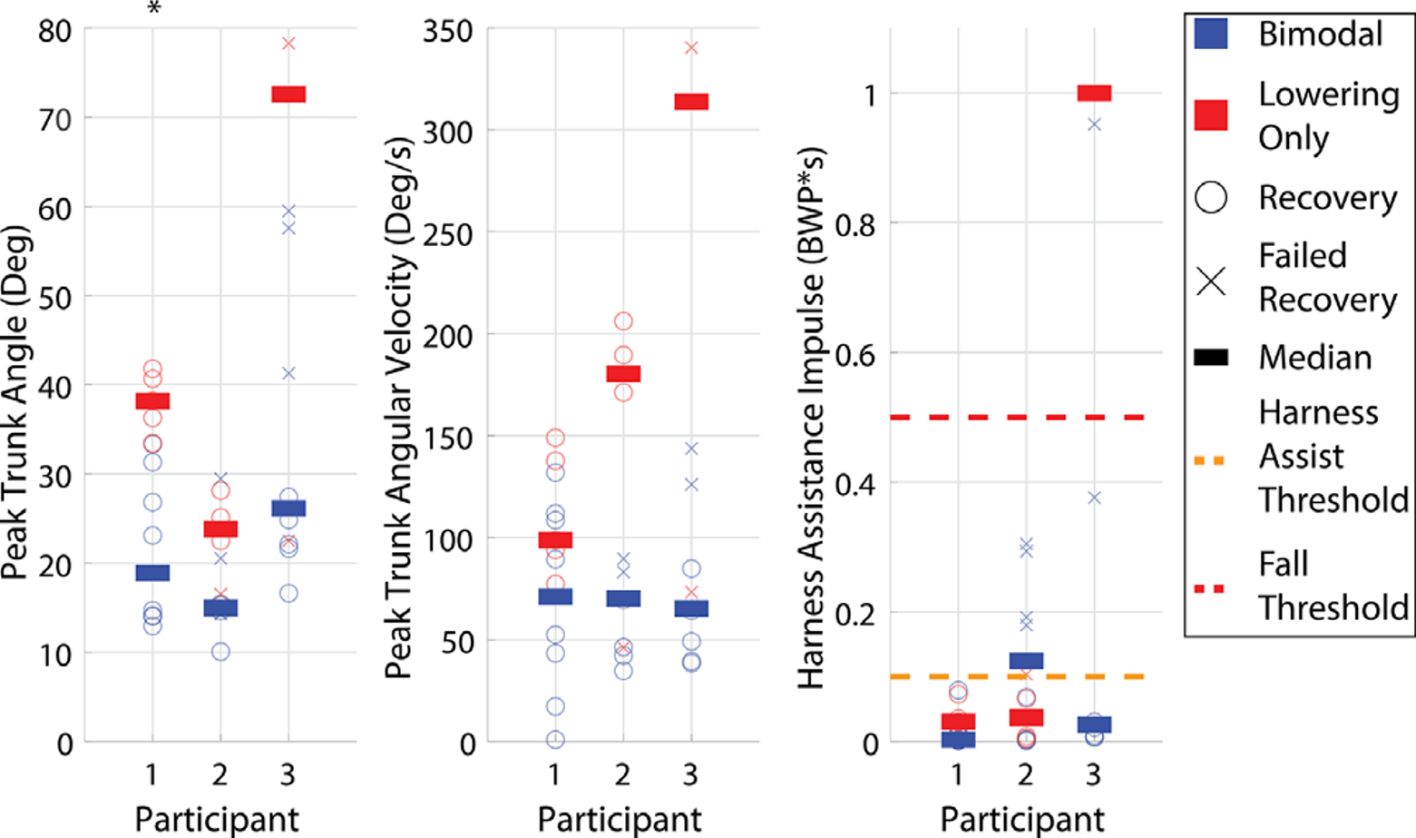
Early swing trunk and harness assistance metrics. Left is the peak trunk angle during the recovery relative to the value at perturbation, middle is the peak trunk angular velocity during the recovery relative to the value at perturbation, and right is the maximum harness assistance impulse during the recovery. The yellow dashed line is the harness assist threshold, and the red dashed line is the fall threshold. Bimodal mode is represented in blue, while Lowering Only mode is represented in red. Empty circles are recoveries, *X*’s are failed recoveries, and the bars are the medians for each mode. Statistical significance between the two controller modes for a given participant is indicated by an asterisk at the top of the column.

**Figure 7.**
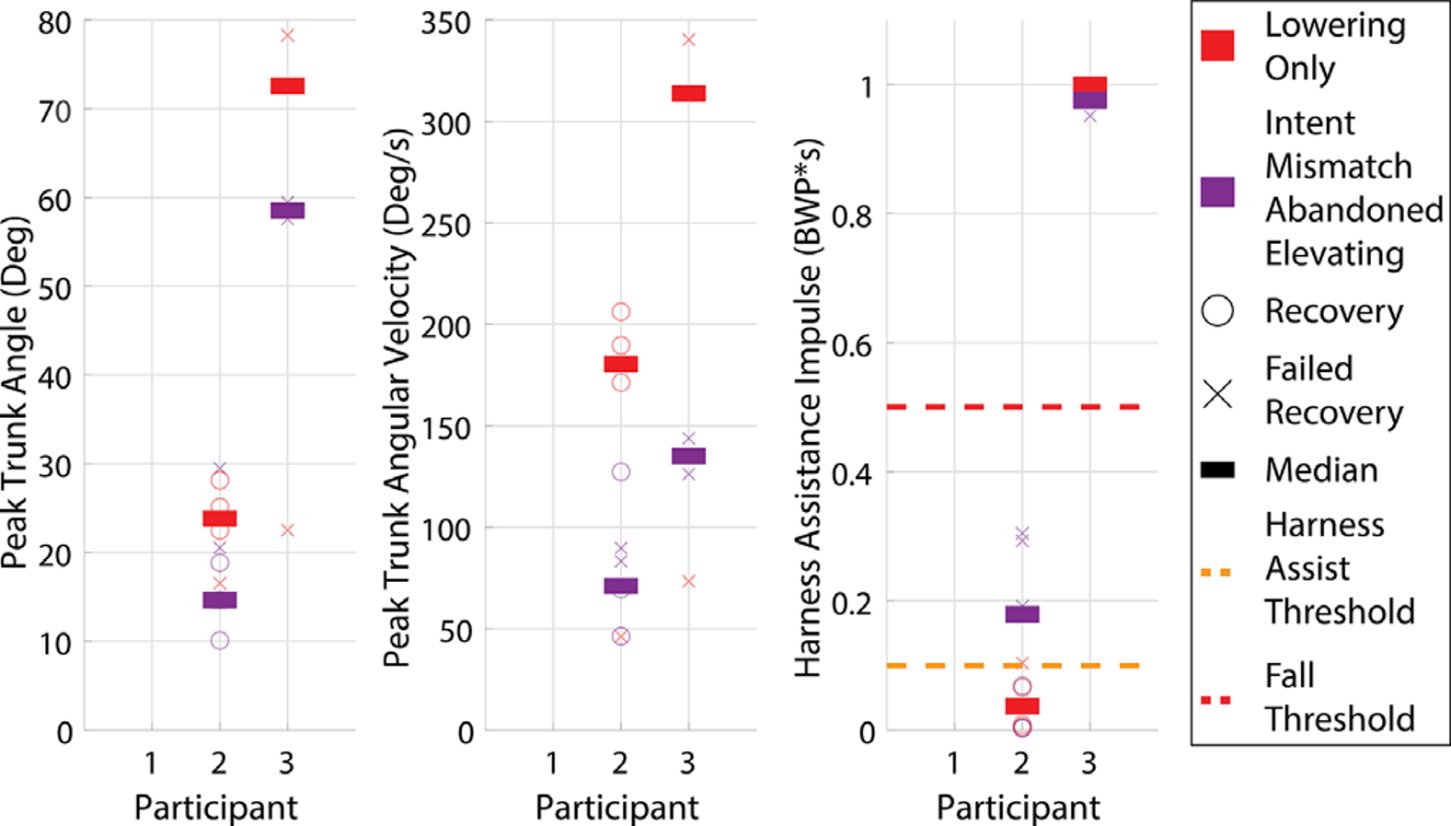
Early swing trunk and harness assistance metrics with intent mismatch (abandoned elevating) responses separated. Left is the peak trunk angle during the recovery relative to the value at perturbation, middle is the peak trunk angular velocity during the recovery relative to the value at perturbation, and right is the maximum harness assistance impulse during the recovery. The yellow dashed line is the harness assist threshold, and the red dashed line is the fall threshold. Abandoned elevating responses while using the Bimodal mode are represented in purple, and the Lowering Only mode is represented in red. Empty circles are recoveries, *X*’s are failed recoveries, and the bars are the medians for each mode.

**Figure 8.**
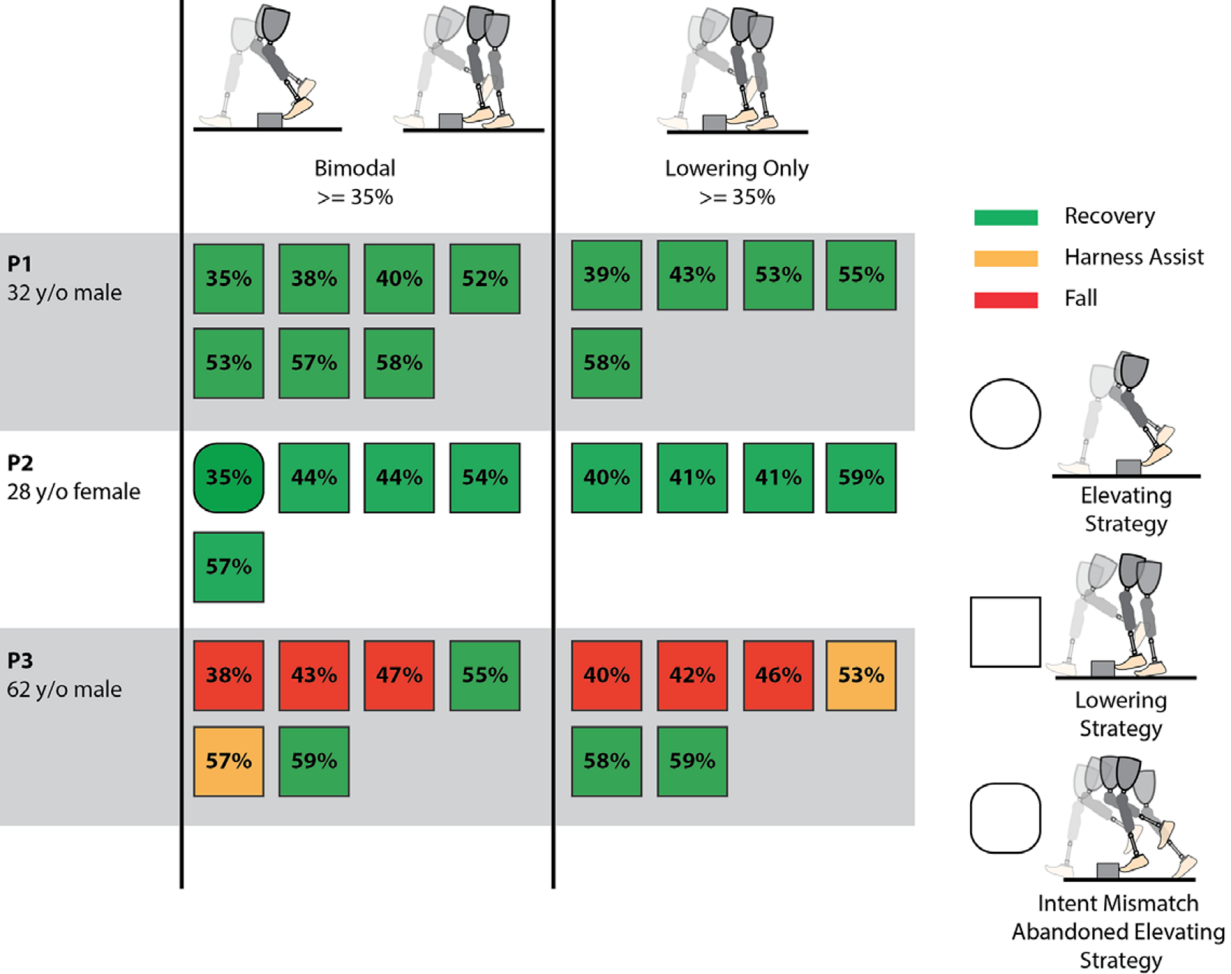
Remainder of recovery outcomes for mid and late swing ( 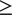 35% swing phase) for each participant and controller. Green indicates a recovery, yellow indicates a harness assist, and red indicates a fall. Elevating strategies are depicted by a circle (not present in this region of swing phase), lowering and delayed lowering strategies are depicted by a square, and intent mismatch trials where the elevating response was abandoned after being chosen by the prosthesis are depicted by rounded squares. The percentage of the swing phase of each perturbation is indicated on the shape.

**Figure 9.**
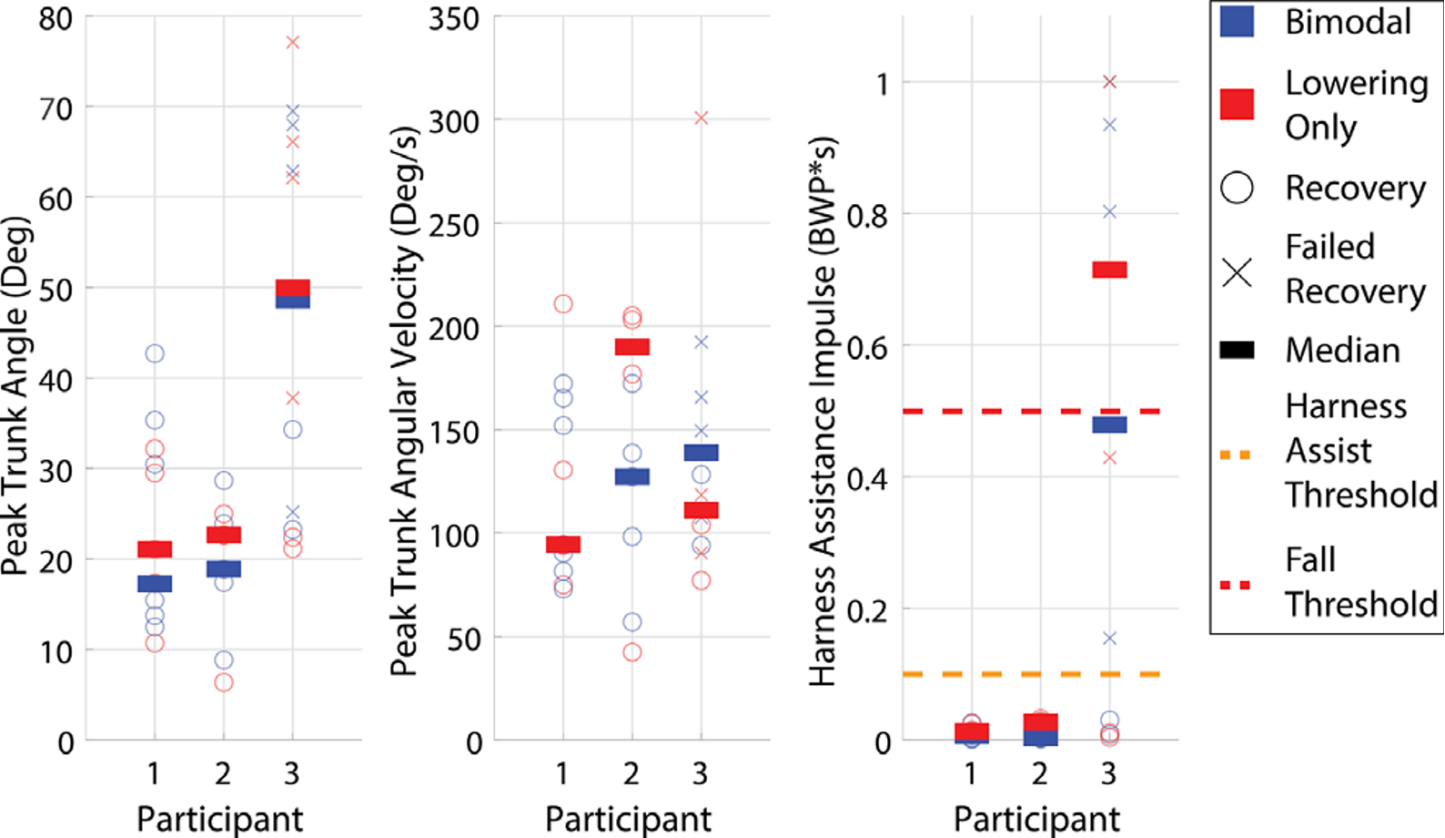
Mid and late swing trunk and harness assistance metrics. Left is the peak trunk angle during the recovery relative to the value at perturbation, middle is the peak trunk angular velocity during the recovery relative to the value at perturbation, and right is the maximum harness assistance impulse during the recovery. The yellow dashed line is the harness assist threshold, and the red dashed line is the fall threshold. Bimodal mode is represented in blue, while Lowering Only mode is represented in red. Empty circles are recoveries, *X*’s are failed recoveries, and the bars are the medians for each mode.

Across the three participants and two controller modes, 69 perturbation trials were collected. All perturbations in all trials were detected by the controller with no false positives (i.e., 100% detection rate). Results corresponding to stumble trials using each of the two controllers (i.e., Bimodal and Lowering Only) are presented separately for early swing (<35% swing phase), and mid to late swing (remainder of swing phase), since early swing is the period in which an elevating response would generally occur in able-bodied individuals. Specifically, results for early swing perturbations are shown in Figures 5–7, while results for mid to late swing perturbations are shown in Figures 8 and 9.

For early swing, based on harness assistance impulse, Participant 1 recovered from all perturbations using both the Bimodal and Lowering Only controllers. Participant 2 recovered from 50% of the perturbations when using the Bimodal controller versus 75% with the Lowering Only controller. Participant 3 recovered from 62.5% of the perturbations using the Bimodal controller, but did not recover from any (0%) with the Lowering Only controller. It is noteworthy that, for Participant 1, while the perturbation at 8% of swing phase was out of the typically targeted range, this occurred due to variability in the predictive targeting algorithm used in the stumble perturbation system. Generally, perturbations this early do not induce a stumble due to swing knee flexion raising the toe over the obstacle; however, in this case, a stumble was induced, so the trial was included in the analysis.

While the Bimodal controller correctly chose the intended strategy based on the collision dynamics, two out of three participants at some point abandoned the elevating strategy and ultimately lowered instead after the controller had initially chosen to elevate (referred to as “intent mismatch” in Figure 5). Participant 2 abandoned the elevating strategy six times during early swing, as well as one time in mid swing at 35% of swing phase (77.8% of perturbations 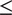 35% swing) with the abandoned elevating resulting in a failed recovery 57.1% of the time (four out of seven perturbations). Participant 3 abandoned the elevating strategy two times during early swing (25% of early swing perturbations), with the abandoned elevating resulting in a failed recovery 100% of the time. It is noteworthy that the variability in recovery strategies (e.g., elevating vs. intent mismatch) across the percentages of swing phase shown in Figure 5 (both within and between participants) supports previous studies demonstrating that the timing of a perturbation in swing phase does not completely predict which recovery strategy will be used (Eveld et al., 2021).

Beyond overall recovery outcomes, trunk kinematic and harness assistance impulse metrics for early swing phase are presented for each participant using each controller in Figure 6. Lower values indicate higher recovery quality or lower likelihood of a fall. Harness assistance impulse values are presented in bodyweight percentage seconds (BWP*s). Values are saturated at 100% bodyweight seconds to avoid skewing the scales by including when participants continued to load the harness for several seconds after a fall.

For all early swing trials, including recoveries, harness assists, falls, and abandoned elevating strategies, the Bimodal controller resulted in lower median peak trunk angle and peak trunk angular velocity for all participants and lower harness assistance impulse for all participants except Participant 2 compared to the Lowering Only controller. The differences in median values were statistically significant for one metric for Participant 1 (trunk angle: 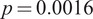 ). While the remainder of the metrics do not demonstrate statistically significant differences (Participant 1’s trunk angular velocity ( 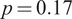 ) and harness assistance impulse ( 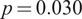 ), all of Participant 2’s trunk angle ( 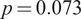 ), trunk angular velocity ( 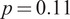 ), and harness assistance impulse ( 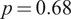 ) and Participant 3’s trunk angle ( 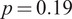 ), trunk angular velocity ( 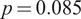 ), and harness assistance impulse ( 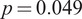 )), a trend exists toward the reduction of all trunk metrics for all participants.

To isolate the impact of the abandoned elevating response (i.e., intent mismatch), Figure 7 presents the recovery metrics of the trials in which the abandoned elevating response occurred (including Participant 2’s trial at 35%) compared to the Lowering Only controller in early swing. The abandoned elevating trunk kinematic metrics and harness assistance impulse medians are similar to or lower than the Lowering Only controller responses for all trunk metrics for all participants and for harness assistance impulse for all participants except Participant 2. None of the metrics for Participant 2 (trunk angle: 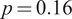 , trunk angular velocity: 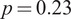 , harness assistance impulse: 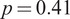 ) demonstrated statistically significant differences. Statistical analysis could not be performed for Participant 3 due to having only two abandoned elevating data points. Therefore, it appears that, in early swing perturbations, when a mismatch occurs between the controller action and the eventual user action, the result is no worse than the Lowering Only response.

The overall recovery outcomes for the remainder of the swing phase 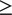 35% (mid to late swing) are presented in Figure 8. Both Participants 1 and 2 recovered from all of the perturbations with both the Bimodal and Lowering Only controllers. Meanwhile, Participant 3 recovered from 33.3% of the perturbations with both the Bimodal and Lowering Only controllers, with the failed recoveries primarily focused around mid swing.

Trunk kinematic and harness assistance impulse metrics for mid to late swing perturbations are presented in Figure 9. The metrics were generally consistent between the two controllers across all three participants. None of the metrics for any participant demonstrated statistically significant differences (Participant 1 (trunk angle: 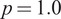 , trunk angular velocity: 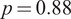 , harness assistance impulse: 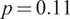 ), Participant 2 (trunk angle: 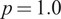 , trunk angular velocity: 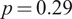 , harness assistance impulse: 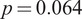 ), or Participant 3 (trunk angle: 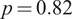 , trunk angular velocity: 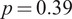 , harness assistance impulse: 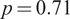 )).

## 4. Discussion

In aggregate, the Bimodal controller improved the quality of the recovery response for all three participants compared to the Lowering Only controller, albeit with limited statistical significance, presumably due to the limited number of data points given the heterogeneity of the experimental conditions. The experimental results are discussed below, particularly with respect to several questions regarding the prospective efficacy of the Bimodal versus the Lowering Only responses, and with respect to the ability of the prosthesis users to coordinate with the different responses.

### 4.1. Can prosthesis users utilize the elevating strategy?

All three participants demonstrated the ability to utilize the elevating strategy with the Bimodal controller to some extent, shown in Figure 5, with two of three (Participant 1 and Participant 3) demonstrating consistent use (i.e., elevating more often than abandoning the elevating response), suggesting the elevating strategy is a feasible, neurologically accessible response for prosthesis users. It is noteworthy that Participant 3 has used a prosthesis for ~50 years; furthermore, Participant 2 has a congenital limb difference and has likely never used the elevating strategy with her affected limb before this study. The response of these participants suggests that with adequate acclimation, the elevating strategy may be used by a wide range of prosthesis users.

Use of the elevating strategy in general is consistent with some prior work with passive prosthetic devices that demonstrated periodic use of the elevating strategy in certain scenarios. One treadmill-based study (Shirota et al., 2015) observed that the elevating strategy was widely used throughout swing phase; however, the use of rope blocking (i.e., no obstacle) and handrails may have affected this outcome, though it corroborates the evidence that the strategy is neurologically accessible. Another treadmill-based obstacle perturbation study (King et al., 2024) observed that the elevating strategy was only used twice by two of the six participants (with only one successful instance) and occurred during stumbles with high prosthetic knee deflection following the perturbation. Therefore, although less commonly observed compared to the rope-blocking study, it also suggests the elevating strategy is neurologically accessible to transfemoral prosthesis users.

### 4.2. Does the elevating strategy provide benefit relative to the lowering strategy in the early swing for prosthesis users?

When comparing the performance of the two controllers, the primary focus is on the early swing phase since that is the region where able-bodied individuals typically employ the elevating strategy, and correspondingly, is the region where prosthesis users would most likely benefit from an elevating response. Prior work has shown that the highest fall rate from treadmill-based obstacle perturbations occurs when using a passive prosthesis in the early swing, potentially due to the inaccessibility of the elevating strategy (King et al., 2024). For early swing, based on overall recovery outcomes alone (i.e., failed recoveries vs. recoveries as defined by harness assistance impulse), both controllers appear to perform similarly; Participant 1 performed equally well with both controllers, Participant 2 performed better with the Lowering Only controller, and Participant 3 performed better with the Bimodal controller. However, assessing the quality of recovery based on continuous measures (i.e., trunk kinematics and harness assistance impulse metrics) suggests differences between the two. Specifically, as demonstrated in Figure 6, the Bimodal controller reduced (i.e., improved) all three outcome metrics for all three participants, except for the harness assistance impulse for Participant 2.

Additionally, while the intent mismatch scenario with the Bimodal controller (Figure 5) often resulted in a failed recovery, there was only one instance where a coordinated elevating strategy resulted in a failed recovery (Participant 3), which was due to a loss of balance several strides after successfully crossing the obstacle. Participant 3 was also wholly unable to successfully utilize the lowering strategy in early swing with the Lowering Only controller, with falls resulting from his foot remaining caught on the obstacle and being unable to initiate the subsequent swing phase in time to recover. During these failed recoveries, Participant 3 demonstrated poor trunk control and poor base of support adjustment with the contralateral limb, which also contributed to the falls. Participant 2 had one instance of a harness assist when using the Lowering Only controller in early swing, which was also the result of the foot remaining caught on the obstacle and failing to initiate the subsequent swing phase while instead resorting to hopping. These results suggest that utilizing the elevating strategy in early swing can be beneficial to the user’s quality of recovery, and therefore could reduce the potential for real-world falls, compared to the lowering strategy.

### 4.3. Can the prosthetic device coordinate with the user’s response to a stumble in a consistent manner?

While all participants were able to utilize the elevating strategy at some point, two of three participants also experienced some difficulty remaining coordinated with the device at least once during the experiment. While the strategy selection is based on the initial movement of the prosthesis after the perturbation, a mismatch in intent between the device and the participant sometimes occurred later in the recovery response after the elevating strategy was initiated. While the collision dynamics were initially well-suited to utilize the elevating strategy, these participants eventually abandoned the elevating strategy in favor of a delayed lowering strategy. A two-phase stumble recovery strategy selection process such as this is consistent with prior work on modeling the recovery strategy selection process in able-bodied individuals (Eveld et al., 2021). While further effort is required to enhance recovery strategy decision algorithm to utilize a two-phase decision scheme to reduce the impact of the abandoned elevating response, some of the control behaviors in the Bimodal controller were already intended to mitigate the effects of the abandoned elevating response, including the short timescale of the elevating response and additional knee flexion in the following stride if the obstacle was not initially cleared. However, the abandoned elevating response was still less effective than in cases when the elevating response was not abandoned. An abandoned elevating response occurred the majority of times the controller selected an elevating response for Participant 2, but only twice for Participant 3. The occurrence of the abandoned elevating response is likely related to the length of time each participant has used a prosthesis and, therefore, has been limited in their ability to utilize the elevating strategy. For example, Participant 3 has used a prosthesis for nearly 50 years, and Participant 2 has a congenital limb difference and likely has never performed the elevating strategy (or at least in an extremely limited manner) before this experiment, possibly limiting her ability to consistently utilize the strategy.

The abandoned elevating responses frequently resulted in failed recoveries. Participant 2’s failed recoveries resulted from the foot remaining caught behind the obstacle due to failure to initiate the subsequent swing phase and resorting to hopping instead. Similarly, Participant 3’s failed recoveries resulted from a failure to initiate the subsequent step, poor base of support adjustment with the contralateral limb, and poor trunk control. However, as seen in Figure 7, the recovery metrics of the intent mismatch cases are consistent with or improved compared to the Lowering Only controller, except for the harness assistance impulse for Participant 2, indicating that the average intent mismatch case still had improved quality of recovery over the Lowering Only controller. Additionally, none of the acclimation period was dedicated to exposing the users to the intent mismatch case, and therefore, their only exposure to it was through inadvertent occurrences. The outcomes for the abandoned elevating response may have improved if these cases were included during the acclimation period or if the decision controller included a case for delayed lowering.

### 4.4. Does having two potential recovery responses lead to increased uncertainty and make recovery more challenging for users?

Both controllers demonstrated similar recovery outcomes as well as trunk and harness assistance metrics for the remainder of swing (i.e., mid to late swing, where both controllers predominantly used the lowering strategy), as shown in Figures 8 and 9. Failed recoveries were proportionally the same across both controllers for all three participants in the remainder of the swing phase, with only Participant 3 experiencing failed recoveries. The failed recoveries were similar to Participant 3’s falls when using the lowering strategy in the early swing phase. While delayed swing initiation still played a role in these falls, the primary issues were poor trunk control and poor base of support adjustments, as Participant 3 would often manage to cross the obstacle following the perturbations later in swing, but his trunk angle was deviated too far to maintain balance.

Beyond the failed recovery rate, the trunk metrics and harness assistance metrics were also consistent across both controllers. The presence of a second possible outcome in the Bimodal controller (i.e., the elevating strategy), therefore, did not appear to change the user’s ability to respond with the lowering strategy later in swing. Therefore, any potential benefits a bimodal approach may provide for early swing recovery seemingly would not adversely affect recovery during mid to late swing.

### 4.5. Implications, future work, and current limitations

Overall, the results suggest that a bimodal response (i.e., employing an elevating response in early swing) may provide benefit to transfemoral prosthesis users, and that, generally, prosthesis users are capable of coordinating with such a bimodal response. Furthermore, the powered stumble recovery behavior eliminated knee buckling observed in prior passive prosthesis stumble studies (Crenshaw et al., 2013b; King et al., 2024), but the assisted swing initiation following the perturbation was not enough to completely prevent participants from remaining caught on the obstacle during the lowering strategy. At a minimum, these results support further exploration of the implementation of the Bimodal controller on a powered prosthesis due to promising recovery outcomes but with limitations in the current implementation and evaluation.

For future evaluation, it is crucial to investigate the impact of the intervention across a larger cohort, including multiple activity levels (i.e., *K*-levels) with an increased number of perturbations for each participant. Ideally, the acclimation period could also be extended to include a longer period of gait training to acclimate to the new strategies, while also allowing the participants to take the device home to acclimate to the powered prosthesis as a whole over an extended period of time in real-world environments. Such an approach would allow for a longitudinal evaluation of the impact of the Bimodal controller as participants acclimate to the elevating strategy over time.

In addition to improving user-device coordination through increased acclimation, it is critical to improve the Bimodal controller’s ability to react to intent mismatch scenarios, or avoid them altogether. One potential solution is the inclusion of a two-phase decision (i.e., first elevating or lowering, then delayed lowering or continue elevating), emulating the delayed lowering response observed in able-bodied individuals (Eveld et al., 2021). The two-phase decision could potentially adjust the response of the device at a faster timescale once the elevating strategy is abandoned. Alternatively, a phase variable control approach during the swing phase could improve the coordination between the device and the user if the elevating strategy is abandoned. By coupling the device’s knee angle to the user’s thigh angle (Rezazadeh et al., 2019), abandoning the elevating strategy would reverse the knee flexion that is initially produced during the early stages of the elevating response.

Beyond reacting to intent mismatch scenarios, an improved understanding of the basis for the reflexive coordination between the user and the device is needed to work toward eliminating the mismatch altogether. The integration of biosignals, such as electromyography, could provide real-time biofeedback to avoid potential mismatch scenarios. Additionally, study of the difference in the muscle activation patterns and timings during the successful elevating responses and the intent mismatch responses may elucidate the cause of the mismatch, whether it be at the reflexive level or the volitional level. In the event of a reflexive response mismatch, the data could be used to refine the decision algorithm based on a better understanding of when these scenarios occur. In the event of a volitional response mismatch, the need for increased acclimation is further supported. Additionally, high-level adaptive behaviors in the control system could support the long-term acclimation to the elevating strategy in a real-world environment. For example, the elevating strategy can be more gradually introduced by initially increasing the bias toward the lowering strategy over the elevating strategy (i.e., raising the configuration space angular offset threshold beyond its current value of 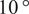 ) and then adaptively reducing the bias as users increase the consistency with which they successfully complete the elevating strategy.

Additionally, further investigation into the impact of phase variable-based swing control (Rezazadeh et al., 2019) is merited to better understand the impact that the overall control architecture has on the stumble recovery decision process. Investigating the strategy selection outcomes when using a temporally-controlled swing trajectory (i.e., the implementation in this paper) compared to a phase variable-controlled (e.g., thigh angle) swing trajectory can help determine if either affects the rate of elevating strategy utilization or intent mismatch occurrence. Furthermore, the investigation would provide insight into the effect of the continued swing trajectory operation during the decision window in the current implementation. The decision to maintain the unperturbed swing trajectory motion while the controller decided on the recovery strategy was intended to avoid imparting potentially disruptive changes in momentum to the user by pausing and resuming knee motion during the perturbation. Additionally, the magnitude of the perturbation’s impact on the dynamics is typically greater than the swing controller’s motion in the 20 ms observation window. However, the continued motion could potentially bias the decision slightly. In particular, in early swing, increased knee flexion could act synergistically with the elevating strategy selection criteria. While the implementation of the 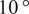 external configuration angle offset when choosing the elevating strategy was partially intended to account for this bias, comparisons to a phase variable-based swing controller would eliminate this temporal coupling without requiring a pause in the trajectory to determine if there is a significant impact on strategy selection or recovery outcomes.

Lastly, decreasing the knee extension velocity during the lowering strategy may also improve the functionality of the proposed controller. In the current implementation, the lowering strategy returns to full knee extension as quickly as possible to ensure maximum knee support to prevent buckling, given the limited amount of time between obstacle contact and ground contact during the lowering strategy. However, the velocity of this response imparts an undesirable angular momentum disturbance to the user, potentially disrupting their CoM control. Reducing knee extension velocity may diminish this undesirable disturbance while maintaining robust knee support during the lowering strategy. Future investigations incorporating these elements will better elucidate the potential benefits of a bimodal versus unimodal stumble recovery response.

## 5. Conclusion

This paper explored the prospective utility of a bimodal versus unimodal stumble recovery response in powered knee prostheses; more specifically, the extent to which a response that decides between an elevating strategy and a lowering strategy might provide benefit relative to one that selects a lowering strategy strictly. Incorporating a decision algorithm described herein, three participants were exposed to similar stumble conditions using both approaches, implemented on an experimental powered prosthesis. All three participants in the study were able to successfully utilize the elevating strategy at some point. When the strategy was used, it generally lowered the disturbance to their trunks and reduced their usage of the harness during recovery in the early swing phase without disturbing their ability to respond to perturbations using the lowering strategy in the mid and late swing phases. Two participants experienced intent mismatch with the device at least once, and one experienced intent mismatch consistently. Although intent mismatch cases (i.e., abandoned elevating) led to less favorable recovery outcomes compared to coordinating with the elevating response, results indicate that it performed at least as well as the Lowering Only approach. Therefore, in this preliminary study, the availability of the elevating strategy improved outcomes when coordinated with the user and maintained outcomes when not coordinated. The overall results suggest that the use of a bimodal response may improve stumble recovery outcomes for prosthesis users.

## Supporting information

10.1017/wtc.2026.10040.sm001 King et al. supplementary movie King et al. supplementary material

## Data Availability

The data that support the findings of this study are available from the corresponding author, STK, upon reasonable request.

## Supplementary material

The supplementary material for this article can be found at http://doi.org/10.1017/wtc.2026.10040.
